# Synthesis and *in vitro* activity of platinum(II) complexes of two fluorenylspirohydantoins against a human tumour cell line

**DOI:** 10.1080/13102818.2014.910363

**Published:** 2014-07-10

**Authors:** Petja Marinova, Marin Marinov, Maria Kazakova, Yana Feodorova, Plamen Penchev, Victoria Sarafian, Neyko Stoyanov

**Affiliations:** ^a^ Faculty of Chemistry, University of Plovdiv, Plovdiv, Bulgaria; ^b^ Faculty of Plant protection and Agroecology, Agricultural University, Plovdiv, Bulgaria; ^c^ Depatment of Biology, Medical University, Plovdiv, Bulgaria; ^d^ University of Ruse, Razgrad, Bulgaria

**Keywords:** fluorenylspirohydantoins, (9′-fluorene)-spiro-5-hydantoin, (9′-fluorene)-spiro-5-(2-thiohydantoin), metal complexes, cytotoxic/antiproliferative effects

## Abstract

This paper presents a method for synthesis and cytotoxicity of new platinum(II) complexes of (9′-fluorene)-spiro-5-hydantoin (L1) and (9′-fluorene)-spiro-5-(2-thiohydantoin) (L2). The new obtained complexes were studied by elemental analysis: ultraviolet–visible, attenuated total reflection Fourier transform infrared (ATR-FTIR), and ^1^H- and ^13^C-NMR for Pt(II) compounds and additionally Raman spectroscopy for free ligands. Based on the experimental data, the most probable structure of the complexes is suggested. In the present study, we have examined cytotoxic activity of (9′-fluorene)-spiro-5-hydantoin (L1) and (9′-fluorene)-spiro-5-(2-thiohydantoin) (L2) and their Pt(II) complexes on the retinoblastoma cell line WERI-Rb-1.

## Introduction

The profound interest on different hydantoin derivatives stems from the well-established medical applications of some hydantoin derivatives as antiepileptic drugs.[1,2] Recently, possible application for HIV-1 therapy has also been suggested.[3,4] In addition, a number of other biological activities of hydantoin derivatives are known as well.[5–8] Fluorene-containing compounds are gaining increasing scientific interest due to their potential application in various optical devices, such as dye-sensitized solar cells,[9] polymer light-emitting diodes (OLED) [10–15] and other electroemissive materials.[16,17] Complexation with metal ions offers different pathways for modulating and improving their optical [18,19] and electrooptical [20,21] properties and therefore is an area of research of significant practical importance. Moreover, there are data for potential application of such compounds as fluorescent sensors for metal ions, such as Cu(II) and Ni(II).[22,23] We have recently described synthetic procedures for formation of thioanalogues of (9′-fluorene)-spiro-5-hydantoin.[24] Moreover, the synthesis and structure of Cu(II) and Ni(II) complexes of (9′-fluorene)-spiro-5-(2,4-dithiohydantoin) have also been studied.[25] In contrast to other spiro-5-(2,4-dithiohydantoins),[26,27] the reaction of the fluorene-substituted 2,4-dithiohydantoin with Cu(II) does not lead to its reduction to Cu(I). The apparent reason for reduction of Cu(II) to Cu(I) is a coordination with the thione sulphur atom, which in the case of (9′-fluorene)-spiro-5-(2,4-dithiohydantoin) is not the preferred one. The crystal structure of (9′-fluorene)-spiro-5-hydantoin (L1) is reported in the literature [28] but there are no X-ray data about its thioanalogues. There are no X-ray data, however, for any metal complexes of (9′-fluorene)-spiro-5-hydantoin or the thioanalogues. That is why, the research described here is focused on Pt(II) complexes of (9′-fluorene)-spiro-5-hydantoin (L1) and (9′-fluorene)-spiro-5-(2-thiohydantoin) (L2) particularly on their structural elucidation and to examine their cytotoxic effect. Several investigations demonstrated that hydantoin derivatives influenced tyrosine kinase activity.[29,30] It has been reported that these compounds could suppress proliferation of lung cancer cells by two pathways, which included inhibiting epidermal growth factor receptor (EGFR) autophosphorylation on the one hand and increasing p53 levels and programmed cell death on the other.[31]

The aim of this study is to present a method for synthesis of new platinum(II) complexes of (9′-fluorene)-spiro-5-hydantoin (L1) and (9′-fluorene)-spiro-5-(2-thiohydantoin) (L2), its isolation in pure form and its characterization using various spectral methods (ultraviolet–visible (UV–vis), attenuated total reflection Fourier transform infrared (ATR-FTIR), and ^1^H- and ^13^C-nuclear magnetic resonance (NMR) spectroscopy) .. Furthermore, considering literature review and biological activity of fluorenylspirohydantoins, the study of the possible effects that these compounds may have on the viability of human tumour cells (WERI-Rb-1) is particularly interesting.

**Figure 1.**
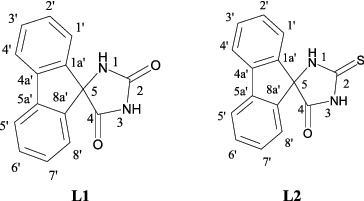
Structural formulas of (9′-fluorene)-spiro-5-hydantoin (L1) and **(**9′-fluorene)-spiro-5-(2-thiohydantoin) (L2).

In the present work, we studied the complexation properties of (9′-fluorene)-spiro-5-hydantoins and its 2-thio derivative (see Figure 1). The reactivity and the structure of the newly obtained platinum(II) complexes were investigated by means of elemental analysis and spectroscopic methods: UV–vis, ATR-FTIR, and ^1^H- and ^13^C-NMR for Pt(II) compounds and additionally Raman spectroscopy for free ligands (L1, L2).

## Materials and methods

A metal salt ((NH_4_)_2_[PtCl_4_] – Sigma-Aldrich) and solvents used for synthesis of the complexes were with a p.a. qualification. The two organic ligands L1 and L2 were synthesized according to methods described in Marinov et al. [24] and Nagasawa et al.[32].

### Synthesis of Pt(II) complex of (9′-fluorene)-spiro-5-hydantoin (L1)

The following were used:

1. 0.0004 mol (0.1001 g) of (9′-fluorene)-spiro-5-hydantoin (L1) in 10 cm^3^ THF;
2. 0.0002 mol (0.0746 g) (NH_4_)_2_[PtCl_4_] in 10 cm^3^ H_2_O;
3. 0.1 m aqueous solution of NaOH in a 100 cm^3^ volumetric flask.

While stirring at pH 10.5 one cubic cm of 0.1 M NaOH was added slowly to an L1 solution. The solution of metal salt was added dropwise from a burette during stirring with electromagnetic stirrer, until precipitation of the formed complex started after 24 h. The complex was formed as brown amorphous precipitate. The precipitate was filtered and washed with 10–20 cm^3^ H_2_O. This was dried over CaCl_2_ for two weeks. It was found that the complex was soluble in DMSO, DMF, CH_3_OH, EtAc, THF, CH_3_COCH_3_ and insoluble in water and C_6_H_6_. The obtained product was in a quantity of 0.0643 g.

### Synthesis of Pt(II) complex of (9′-fluorene)-spiro-5-(2-thiohydantoin) (L2)

The following were used:

1. 0.0004 mol (0.065 g) of (9′-fluorene)-spiro-5-(2-thiohydantoin) (L2) in 10 cm^3^ THF;
2. 0.0002 mol (0.0746 g) (NH_4_)_2_[PtCl_4_] in 10 cm^3^ H_2_O;
3. М aqueous solution of NaOH in a 100 cm^3^ volumetric flask.

A total of 6 cm^3^ of 0.1 M NaOH was added slowly to an L2 solution while stirring at pH 12.5. The solution of metal salt was added dropwise from a burette during stirring with electromagnetic stirrer, until precipitation of the formed complex started after 24 h. A neutral complex was formed as yellow precipitate. The last was filtered and washed with 10–20 cm^3^ H_2_O. This was dried over CaCl_2_ for two weeks. It was found that the complex was soluble in DMSO, DMF and insoluble in water, CH_3_OH, EtAc, C_6_H_6_. The obtained quantity was 0.0520 g.

Electronic spectra of absorption were registered on a Lambda 9 Perkin-Elmer UV/vis/NIR(Near infrared) Spectrophotometer from 200 nm to 1000 nm. ATR-FTIR spectra were registered on a VERTEX 70 spectrometer (Bruker Optics) from 4000 to 600 cm^−1^ at a resolution of 2 cm^−1^ with 25 scans. The ATR accessory was MIRacle™ with a one-reflection ZnSe element (Pike) and the stirred crystals of L1, L2, Pt(II)L1 and Pt(II)L2 were pressed by an anvil to the reflection element. The Raman spectra of L1 and L2 (the stirred crystals placed in aluminium disc) were measured on a RAM II (Bruker Optics) with a focused laser beam of 500 mW power of Nd:YAG laser (1064 nm) from 4000 to 100 cm^−1^ at a resolution of 2 cm^−1^ with 25 scans. The NMR spectra were taken on a Bruker Avance II+ 600MHz NMR spectrometer operating at 600.130 and 150.903 MHz for ^1^H and ^13^C, respectively, using the standard Bruker software. Chemical shifts were referenced to tetramethylsilane (TMS). Measurements were carried out at ambient temperature.

The physicochemical and spectral characterizations of the obtained compounds were as follows:

**Table:** 

**Elemental analysis for L1**:
Calculated: [C_15_H_10_N_2_O_2_] (%): C, 72.0; H, 4.0; N, 11.2;
Determined: C, 72.0; H, 4.3; N, 11.3.
**Elemental analysis for Pt(II)L1** Mw(Pt(II)L1) = 1694.57 g/mol:
Calculated: [C_90_H_58_N_12_O_12_Pt] (%): C, 63.9; H, 3.2; N, 9.9
Determined: C, 64.6; H, 3.4; N, 9.7.
**UV*–*vis (DMSO) L1: λ_max_** = 215, 222, 224, 231 and 275 nm.
**UV–vis (DMSO) Pt(II)L1: λ_max_** = 227 and 276 nm.
**ATR (ν_max_, cm^−1^): L1:** 3360 (ν_NH_), 3189 (ν_NH_), 3075 (ν_CH_), 3009 (ν_CH_), 2721, 1775 (ν_CO_), 1715 (ν_CO_), 1654, 1602, 1585, 1566, 1477, 1450, 1397, 1289, 1235, 1204, 1156, 1116, 1083, 1015, 990, 976, 934, 925, 773, 755, 735, 722, 683, 652, 638, 620, 605.
**ATR (ν_max_, cm^−1^): Pt(II)L1**: 3232 (ν_NH_), 3136 (ν_NH_), 3046 (ν_CH_), 2756, 1774 (ν_CO_), 1705 (ν_CO_), 1605, 1585, 1474, 1449, 1434, 1386, 1289, 1228, 1209, 1153, 1122, 1078, 1043, 980, 947, 925, 875, 788, 763, 756, 743, 730, 683, 658, 639, 619, 612.
**Raman (ν_max_, cm^−1^): L1**: 3073 (ν_CH_), 3041 (ν_CH_), 3012 (ν_CH_), 1777 (ν_CO_), 1757, 1605, 1584, 1486, 1450, 1362, 1351, 1299, 1230, 1154, 1115, 1084, 1023, 990, 785, 736, 724, 684, 638, 514, 445, 414, 330, 311, 280, 255, 183, 169, 124.
^1^H NMR (δ/ppm, multiplicity, DMSO-d_6_) L1: 11.26 (s, NH-3), 8.61 (s, NH-1), 7.89 (d, 4′/5′), 7.51–7.47 (m, H-1′/8′, H-3′/6′), 7.37 (t, H-2′/7′).
^1^**H NMR (δ/ppm, multiplicity, DMSO-d_6_) Pt(II)L1**: 8.59 (s, NH-1), 7.89 (d, 4′/5′), 7.51–7.46 (m, H-1′/8′, H-3′/6′), 7.37 (t, H-2′/7′).
^13^**C NMR (δ/ppm, DMSO-d_6_) L1:** 174.59 (C_4_ = O), 158.06 (C_2_ = O), 143.38 (C_1a’/8a’_), 141.12 (C_4a’/5a’_), 130.28 (CH_3’/6’_), 128.81 (CH_2’/7’_), 124.02 (CH_1’/8’_), 121.20 (CH_4’/5’_), 72.84 (C_5_).
^13^**C NMR (δ/ppm, DMSO-d_6_) Pt(II)L1**: 174.56 (C_4_ = O), 158.02 (C_2_ = O), 143.36 (C_1a’/8a’_), 141.10 (C_4a’/5a’_), 130.26 (C_3’/6’_H), 128.79 (C_2’/7’_H), 124.00 (C_1’/8’_H), 121.18 (C_4’/5’_H), 72.81 (C_5_).
**Elemental analysis for L2**:
Calculated: [C_15_H_10_N_2_OS] (%): C, 67.7; H, 3.8; N, 10.5; S, 12.0;
Determined: C, 67.4; H, 3.9; N, 10.6; S, 12.3.
**Elemental analysis for Pt(II)L2 Mw(Pt(II)L2) = 725.70 g/mo**l:
Calculated: [C_30_H_18_N_4_S_2_O_2_Pt] (%): C, 49.7; H, 2.5; N, 7.7;
Determined: C, 49.2; H, 2.2; N, 7.8.
**UV–vis (DMSO) L2: λ_max_** = 216, 225 and 275 nm.
**UV–vis (DMSO) Pt(II)L2: λ_max_** = 222, 230 and 273 nm.
**ATR (ν_max_, cm^−1^): L2**: 3244 (sh., ν_NH_), 3153 (ν_NH_), 3091 (ν_CH_), 2909, 1751 (ν_CO_), 1729 (ν_CO_), 1606, 1530 (ν_CS_), 1503, 1475, 1449, 1401, 1374, 1288, 1249, 1191, 1167, 1150, 1110, 1072, 1006, 944, 923, 879, 866, 793, 775, 756, 740, 733, 724, 711, 675, 657, 636, 625, 619.
**ATR (ν_max_, cm^−1^): Pt(II)L2**: 3064, 2867, 1772 (ν_CO_), 1752 (ν_CO_), 1635, 1527, 1475, 1449, 1386, 1290, 1264, 1216, 1182, 1108, 1065, 1032, 1021, 1004, 943, 922, 871, 758, 734, 677, 641, 629, 620.
**Raman (ν_max_, cm^−1^): L2**: 3067 (ν_CH_), 3050 (ν_CH_), 1728 (ν_CO_), 1624, 1607, 1486, 1448, 1359, 1296, 1233, 1211, 1157, 1151, 1113, 1074, 1022, 1006, 942, 786, 760, 743, 726, 675, 624, 537, 515, 475, 418, 362, 296, 271, 254, 165, 115.
**^1^H NMR (δ/ppm, multiplicity, DMSO-d_6_) L2**: 12.36 (s, NH-3), 10.61 (s, NH-1), 7.90 (d, H-4′/5′), 7.48 (td, H-3′/6′), 7.39 (d, H-1′/8′), 7.36 (t, H-2′/7′).
^13^**C NMR (δ, ppm, DMSO-d_6_) L2**: 183.52 (C_4_ = O), 174.72 (C_2_ = S), 141.31 (C_1a’/8a’_), 140.71 (C_4a’/5a’_), 130.19 (C_3’/6’_H), 128.56 (C_2’/7’_H), 123.68 (C_1’/8’_H), 120.98 (C_4’/5’_H), 74.75 (C_5_).

### Biological assay

#### Cell line

The retinoblastoma cell line (WERI-Rb1) was purchased from ATCC-HTB-169. It was cultured in RPMI 1640, containing 10% FCS, streptomycin and penicillin at 37 ºC in a humidified atmosphere containing 5% CO_2_.

### WST-1 assay and cytotoxicity

The cytotoxic effect of L1, L2, Pt(II)L1 and Pt(II)L2 was assessed on a retinoblastoma cell line using WST-1 assay (Cat. No11 644 807 001, Roche). All compounds were first dissolved in DMSO and then diluted in the culture medium. The concentration of DMSO in the wells did not exceed 1%. Cells were seeded in triplicates in 96-well plates at a density of 6.5 × 10^4^ cells/well. After a cultivation period of 24 h, the compounds were added at a concentration of 100 μM and incubated for 24, 48 and 72 h, respectively. WST-1 was added to the cells at these time points and incubated for 4h. WST-1 is a colorimetric assay for the non-radioactive quantification of cell proliferation, viability and cytotoxicity. The highest achievable concentration at which we were able to test it was only 100 μM.

Absorbance was measured on ELISA SUNRISE Reader. The wavelength for measuring the absorbance of the formazan product is 450 nm and the reference filter was set at 620 nm in accordance with the WST-1 manual. Cells grown in culture medium alone and in appropriate concentrations of DMSO were used as controls. The percentage of viable cells was calculated as a ratio of the OD value of the sample to the OD value of the control. The data are presented as mean ± standard error of the mean.

## Results and discussion

Complexation with Pt(II) was conducted under alkaline conditions using a metal salt namely (NH_4_)_2_[PtCl_4_] at molar ratio M:L:OH^−^ = **2:4:1** for **PtL1** and **1:2:3** for **PtL2**. Neutral complexes were synthesized and isolated as brown and yellow precipitates, respectively. All complexes were investigated by elemental analysis: UV–vis, ATR-FTIR, and ^1^H- and ^13^C-NMR spectroscopy.

The elemental analyses data showed metal-to-ligand ratio 1:6 for PtL1 and 1:2 for PtL2. The UV–vis spectra were registered in DMSO. Maxima in the UV–vis spectra of the free ligands L1 and L2 were observed at λ_max_ = 215, 222, 224, 231, 275 nm and λ_max_ = 216, 225, 275 nm, respectively. Maxima in the electronic spectra of Pt(II)L1 and Pt(II)L2 complexes were observed at 227 nm, 276 nm and 222 nm, 230 nm, 273 nm, respectively. Solid ATR-FTIR spectra of the free ligand and its complexes were recorded in order to clarify the structure of the formed metal complexes and to determine the coordination modes of the ligand. All vibrational frequencies observed in the ATR-FTIR spectra of the complexes and the free ligands were given in “Materials and Methods” section. The bands at 3360 and 3189 cm^−1^ of the free ligand L1 may refer to the stretching vibrations of two N–H groups of the hydantoin ring. In the ATR spectrum of the PtL1 complex, the same bands were observed at 3232 and 3136 сm^−1^ which shifts to lower frequency by 128 and 53 сm^−1^ as compared to the corresponding free ligand bands. The bands of free ligand L1 at 1775 and 1715 сm^−1^ can be attributed to the stretching vibration of C^4^ = O and C^2^ = O groups of the hydantoin ring. The same bands in ATR spectrum of PtL1 complex were at 1774 and 1705 сm^−1^, i.e. a slight change is observed. This fact shows that the two carbonyl groups of the ligand hydantoin ring did not participate in the coordination.

In the ATR spectrum of the free ligand L2, the bands at 3244 cm^−1^ (shoulder) and 3153 cm^−1^ were observed which may refer to the stretching vibrations of N–H groups of the hydantoin ring. These bands disappeared in the spectrum of the PtL2 complex; there was a very wide, low-intensity band between 3500 and 2500 cm^−1^. On the whole, all bands in the ATR spectrum of PtL2 complex were wider than those in the free ligand. In the spectrum of L2, there were bands at 1751 and 1729 сm^−1^ and 1530 сm^−1^; the first two bands can be attributed to the split stretching vibration of C^4^ = O, and the second to stretching vibration of C^2^ = S group. For the PtL2 complex, the first two bands were shifted by 21 and 23 сm^−1^ to 1772 and 1752 сm^−1^, respectively. On the other hand, the thiocarbonyl stretching was shifted by only 3–1527 сm^−1^. This fact shows that the thiocarbonyl group did not participate in the coordination with metal ion. Based on the experimental data, the most probable structure for the PtL1 complex was suggested with two deprotonated NH groups of ligand L1 and four uncharged organic ligands molecules. For the PtL2 complex square planar geometry was suggested with two ligand molecules coordinated in a bidentate fashion.

**Figure 2.**
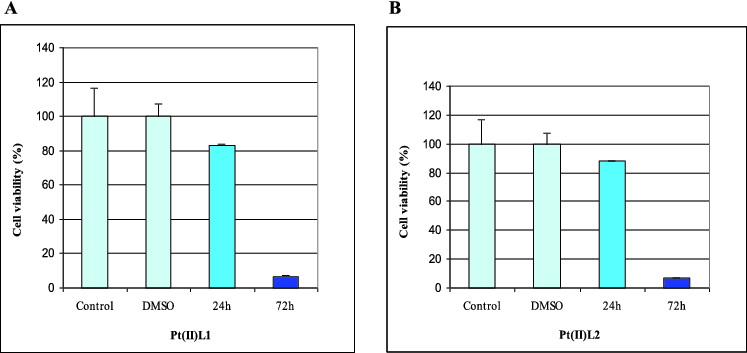
Effect of Pt(II)L1 (A) and Pt(II)L2 (B) on proliferation measured by WST-1assay at 24 and 72 h of treatment.

**Figure 3.**
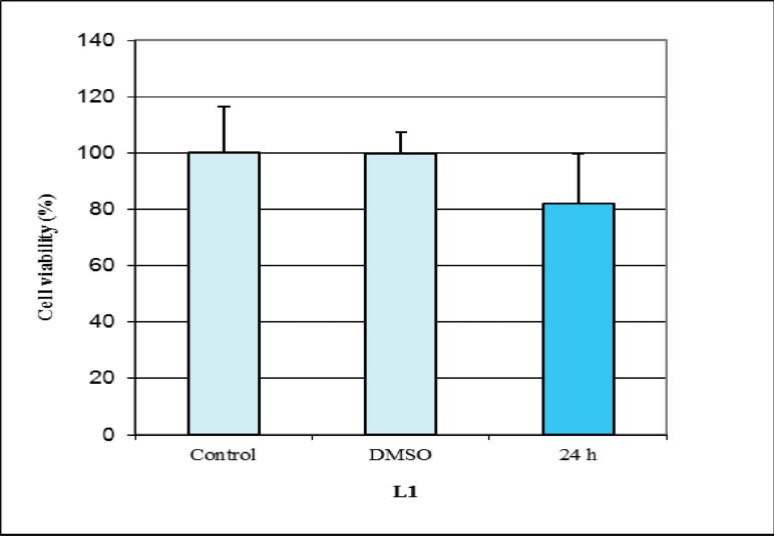
Effect of (9′-fluorene)-spiro-5-hydantoin (L1) on proliferation measured by WST-1 assay at 24 h of treatment.

Our results revealed that L1, Pt(II)L1 and Pt(II)L2 had a cytotoxic effect on the human retinoblastoma cell line WERI-Rb1 (Figures 2 and  3). We found that prolonged incubation periods dramatically influenced cell viability. The Pt(II)L1 and Pt(II)L2 complexes showed significant effects on cancer cell growth compared to their ligands. The data from the cytotoxicity assay showed that Pt(II)L1 and Pt(II)L2 reduced the number of cells by around 20% after 24 h and led to complete inhibition of cell viability at 72 h of treatment (Figure 2). It may be speculated that the presence of Pt(II) ion might be responsible for the cytotoxic properties of the complexes. The ligand L2, in contrast, did not show any inhibitory activity within the tested concentration, while L1 reduced the amount of cancer cells by around 20% after 24 h only (Figure 3). We did not find any activity at 48 h of treatment. Summarized data of cell viability are presented in Table 1.

**Table 1.** Cell viability of WERI-Rb1 cells assessed by WST-1 assay.

Compound	Treatment period	% viability*
L1	24–72 h	81.82 ± 18
Pt(II)L1	24–72 h	83.36 ± 0.41 6.77 ± 0.27
L2	24–72 h	–
Pt(II)L2	24–72 h	88.26 ± 0.2 6.86 ± 0.2

*Values represent mean ± SD.

Based on these results further *in vitro* experiments are required to study the possible applications of hydantoin derivatives and their mechanism of action.

## Conclusions

The synthesis of two Pt(II) complexes with (9′-fluorene)-spiro-5-hydantoin (L1) and (9′-fluorene)-spiro-5-(2-thiohydantoin) (L2) were described and the various spectral data, UV–vis, ATR-FTIR, and ^1^H- and ^13^C-NMR spectroscopy, confirmed their structures. The preliminary results of our study showed that L1, Pt(II)L1 and Pt(II)L2 exhibit anticancer activity *in vitro*.
